# Supplementary material for: Differential Proliferation Effect of the Newly Synthesized Valine, Tyrosine and Tryptophan–Naphthoquinones in Immortal and Tumorigenic Cervical Cell Lines
Source: Molecules. 2020 Apr 28;25(9):2058. doi: 10.3390/molecules25092058 (PMC7248809; doi:10.3390/molecules25092058)
Supplement: Supplementary file 1 [file molecules-25-02058-s001.pdf]

Supplementary data

Differential Proliferation Effect of the Newly Synthesized Valine, Tyrosine and Tryptophan–Naphthoquinones in Immortal and Tumorigenic Cervical Cell Lines

Sergio Córdova-Rivas [1], Jorge Gustavo Araujo-Huitrado [1], Ernesto Rivera-Avalos [2], Ismailia L. Escalante-García [3], Sergio M. Durón-Torres [3], Yamilé López-Hernández [4], Hiram Hernández-López [3], Lluvia López [5] Denisse de Loera [2,*] and Jesús Adrián López [1,*]

[1] Laboratorio de microRNAs y Cáncer, Unidad Académica de Ciencias Biológicas, Universidad Autónoma de Zacatecas, ZAC 98068, México; cordova092@hotmail.com (S.C.-R); aahj011871@uaz.edu.mx (J.G.A.-H.)

[2] School of Chemistry, Universidad Autónoma de San Luis Potosí, SLP 78210, México; neto_riava@hotmail.com

[3] Unidad Académica de Ciencias Químicas, Universidad Autónoma de Zacatecas, ZAC 98160, México; ileg@uaz.edu.mx (I.L.E.-G.); duronm@uaz.edu.mx (S.M.D.-T.); hiram.hernandez.lopez@uaz.edu.mx (H.H.-L.)

[4] CONACYT, Laboratorio de Metabolómica y Proteómica, Unidad Académica de Ciencias Biológicas, Universidad Autónoma de Zacatecas, Zac 98068, México; ylopezher@conacyt.mx

[5] Instituto de Investigación de Zonas Desérticas, Universidad Autónoma de San Luis Potosí, SLP 78377, México; lluvia.lopez@uaslp.mx

* Correspondence: atenea.deloera@uaslp.mx (D.d.L.); jalopez@uaz.edu.mx (J.A.L.); Tel.: +52-444-826-2300 x6415 (D.d.L.); +52-492-149-2648 (J.A.L.)

CONTENT

1. Infrared characterization 2
   Amino acids-1,4-naphthoquinone derivatives: 2
   Amino acids-2,3-dichloronaphthoquinone derivatives: 3
2. NMR characterization 5
   Amino acids-1,4-naphthoquinone derivatives: 5
   Amino acids-2,3-dichloronaphthoquinone derivatives: 8
3. Mass spectrometry characterization 11
   Amino acids-1,4-naphthoquinone derivatives: 11
   Amino acids-2,3-dichloronaphthoquinone derivatives: 12
4. EPR characterization 14
   Compound 2-((1,4-dioxo-1,4-dihydronaphthalen-2-yl)amino)-4-(methylthio)butanoic acid 14

1. Infrared Characterization

Amino acids-1,4-naphthoquinone derivatives:

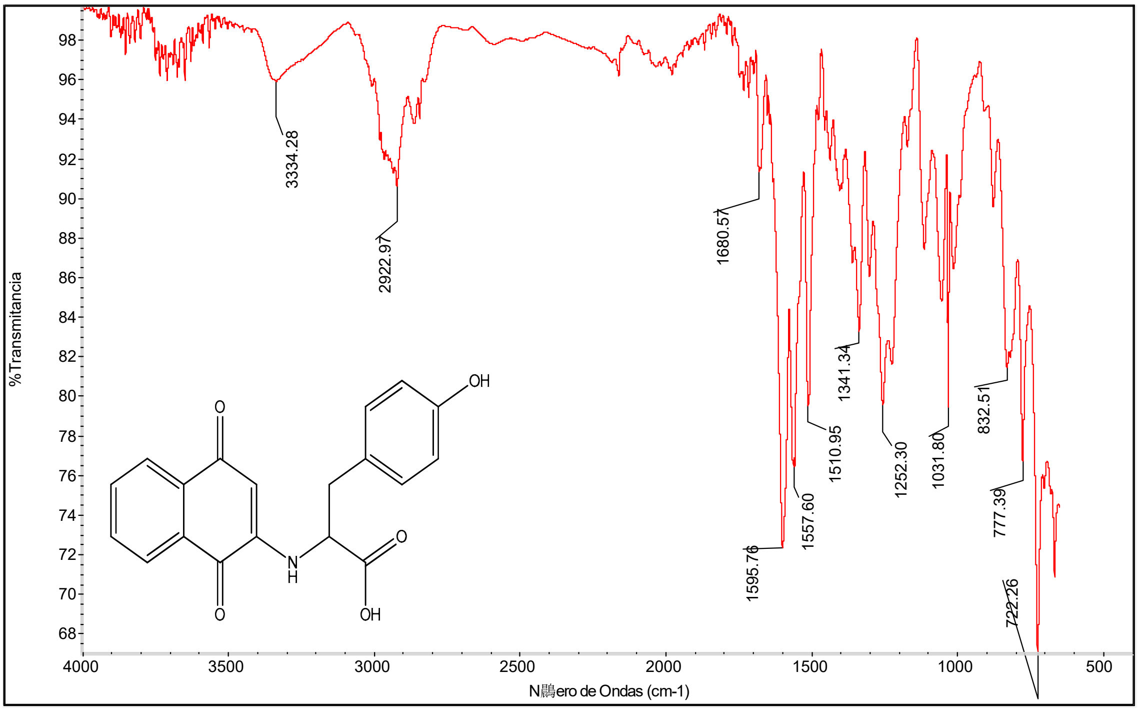

Figure S1. IR spectrum of 2-((1,4-dioxo-1,4-dihydronphthalen-2-yl)amino)-3-(4-hydroxyphenyl) propanoic acid (3a).

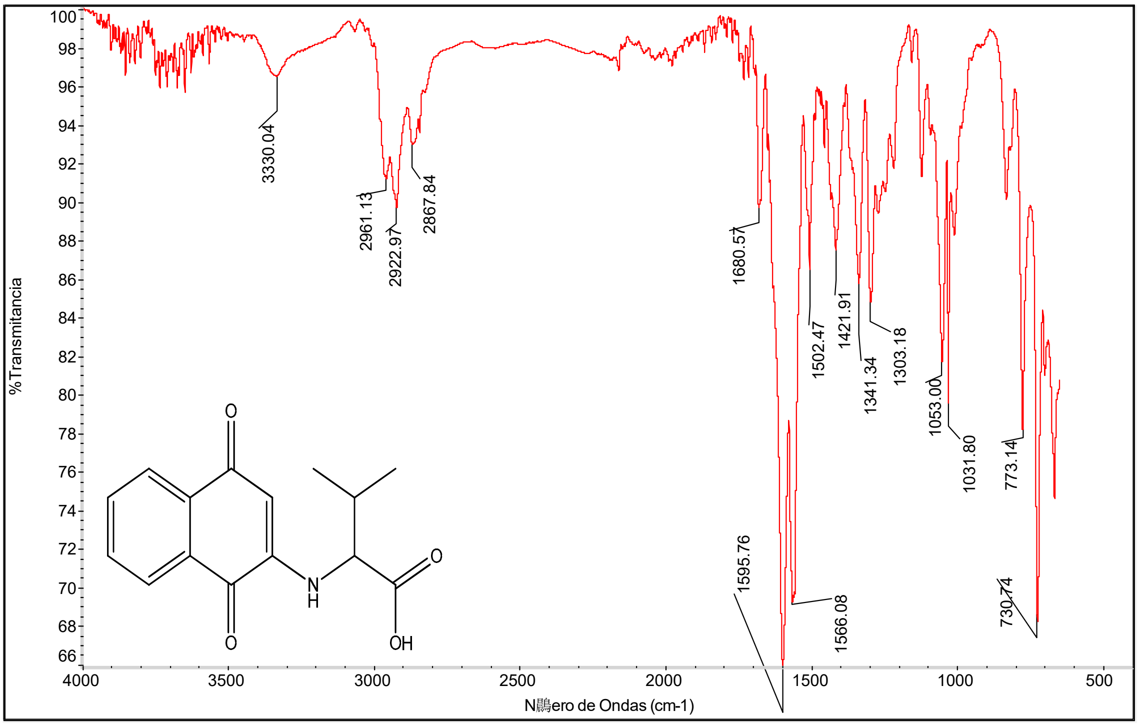

Figure S2. IR spectrum of 2-((1,4-dioxo-1,4-dihydronaphthalen-2-yl)amino)-3-methylbutanoic acid (3b).

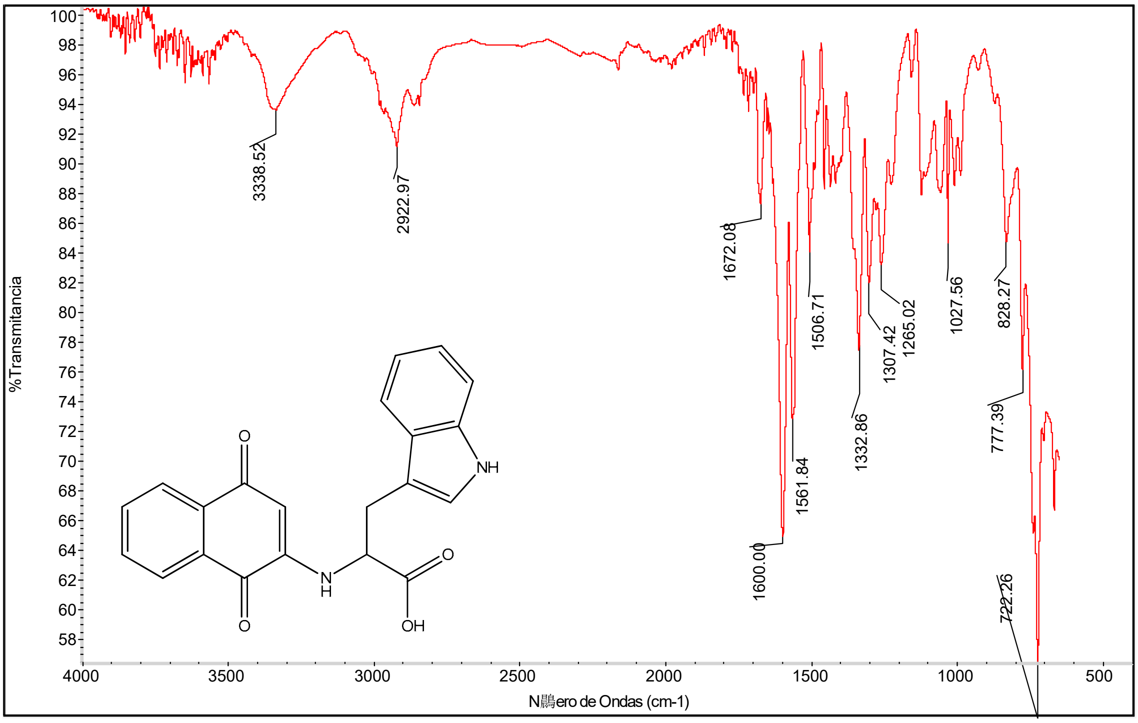

Figure S3. IR spectrum of 2-((1,4-dioxo-1,4-dihidronaphthalen-2-yl)amino)-3-(1*H*-indol-3-yl)propanoic acid (3c).

Amino acids-2,3-dichloronaphthoquinone derivatives:

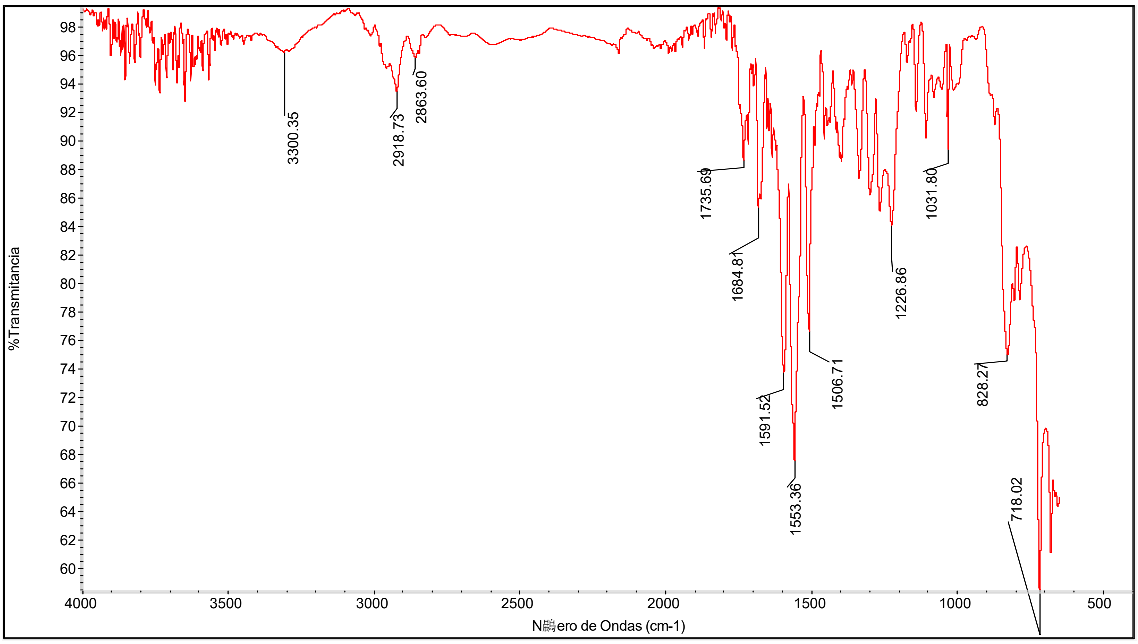

Figure S4. IR spectrum of 2-((3-chloro-1,4-dioxo-1,4-dihydronaphthalen-2-yl)amino)-3-(4-hydroxyphenyl)propanoic acid (4a).

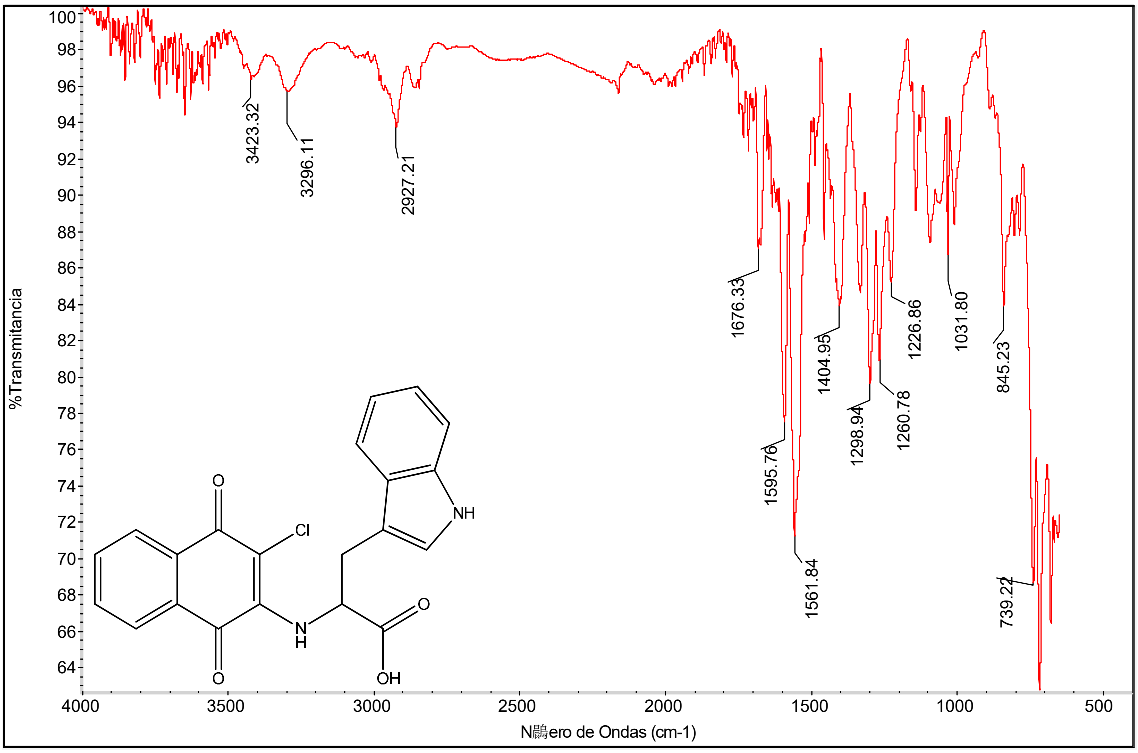

Figure S5. IR spectrum of 2-((3-chloro-1,4-dioxo-1,4-dihydronaphthalen-2-yl)amino)-3-methylbutanoic acid (4b).

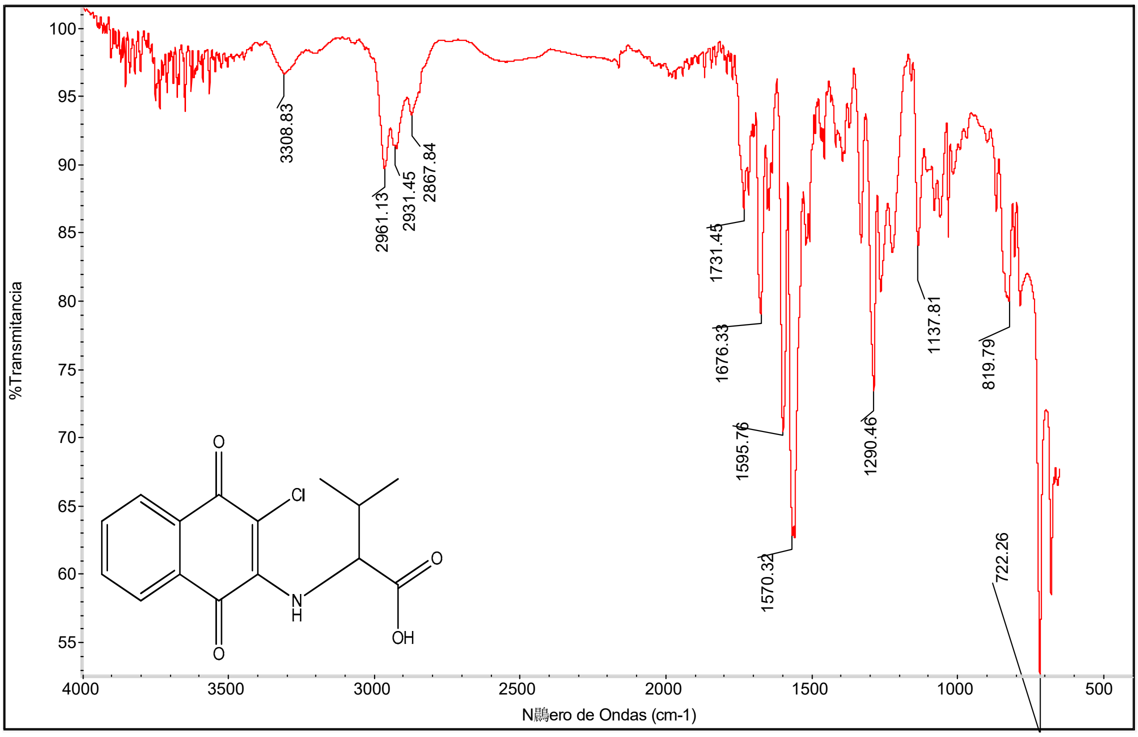

Figure S6. IR spectrum of 2-((3-chloro-1,4-dioxo-1,4-dihydronaphthalen-2-yl)amino)-3-(1H-indol-3-yl)propanoic acid (4c).

2. NMR Characterization

Amino acids-1,4-naphthoquinone derivatives:

A

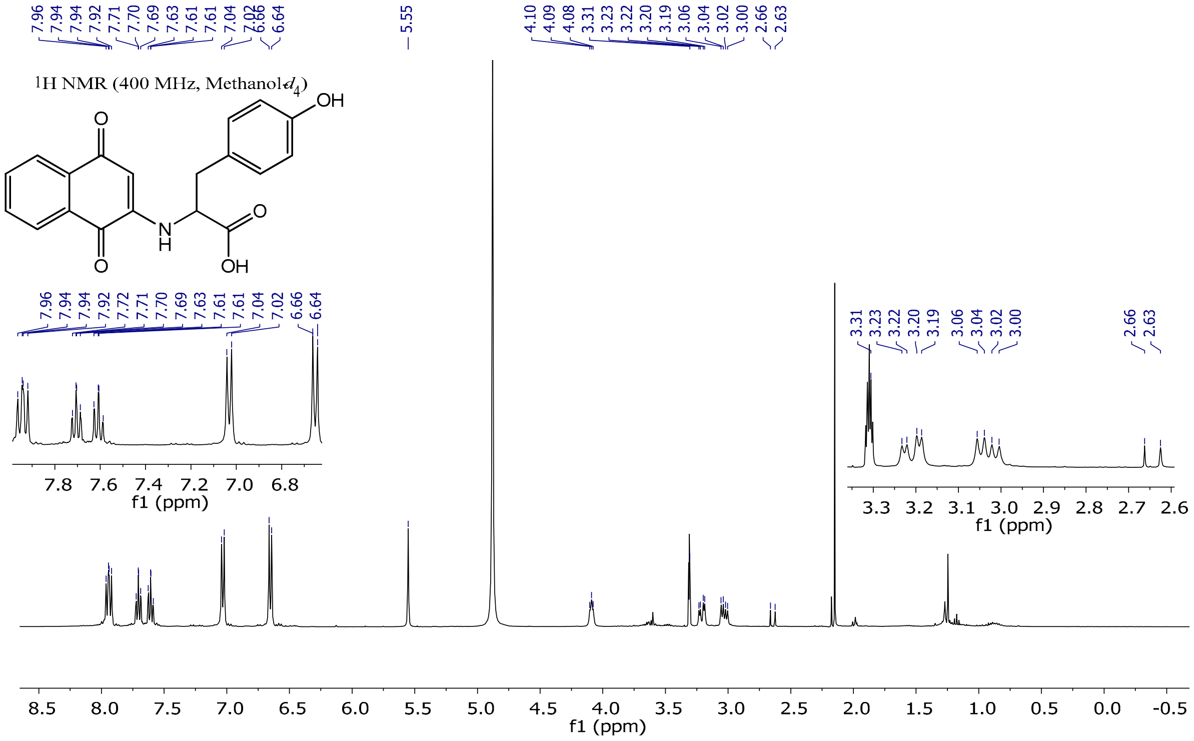

B

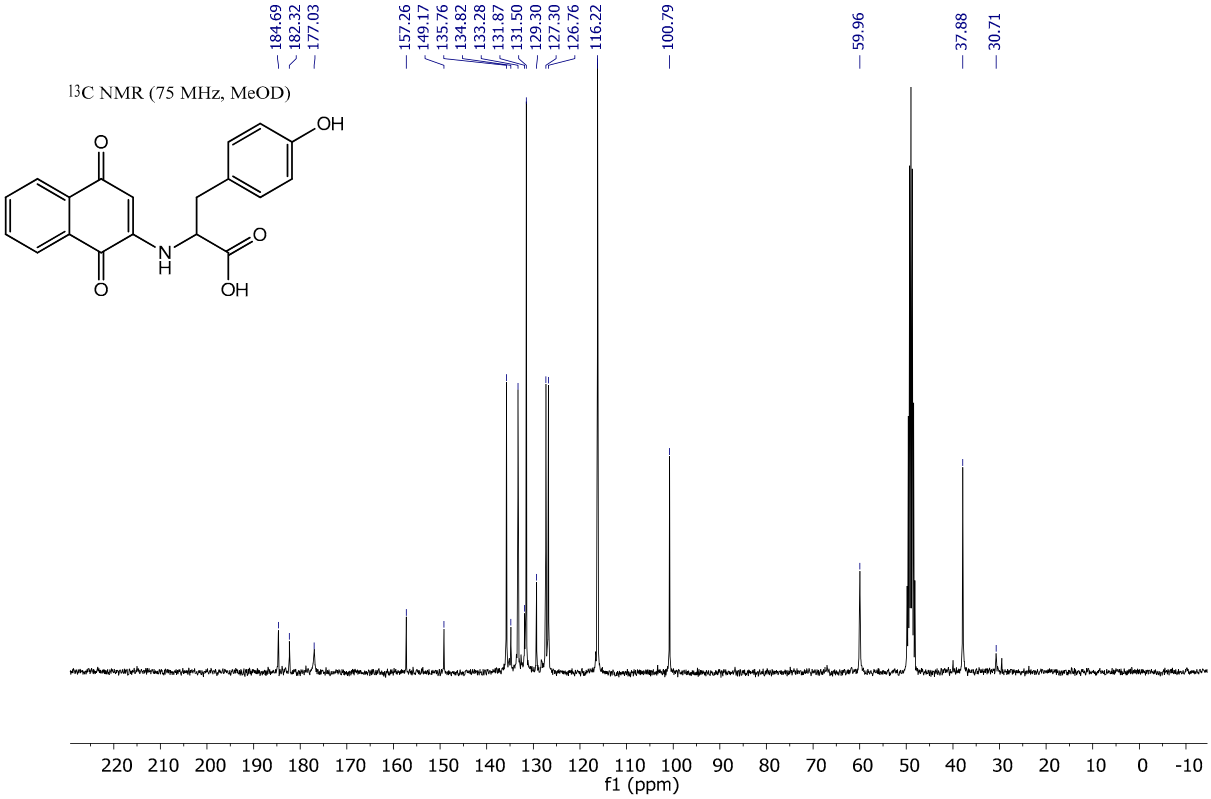

Figure S7. NMR spectrum of 2-((1,4-dioxo-1,4-dihydronphthalen-2-yl)amino)-3-(4-hydroxyphenyl) propanoic acid (3a), ^{1}H (**A**) and ^{13}C (**B**).

A

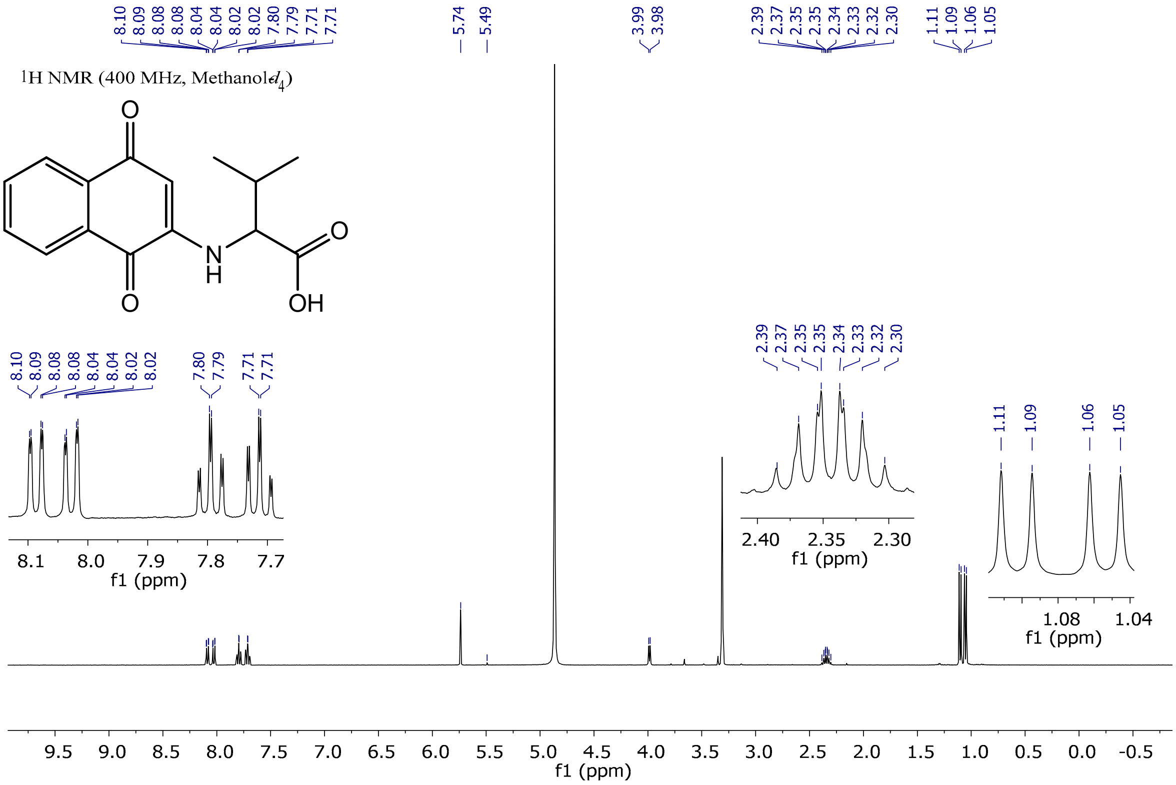

B

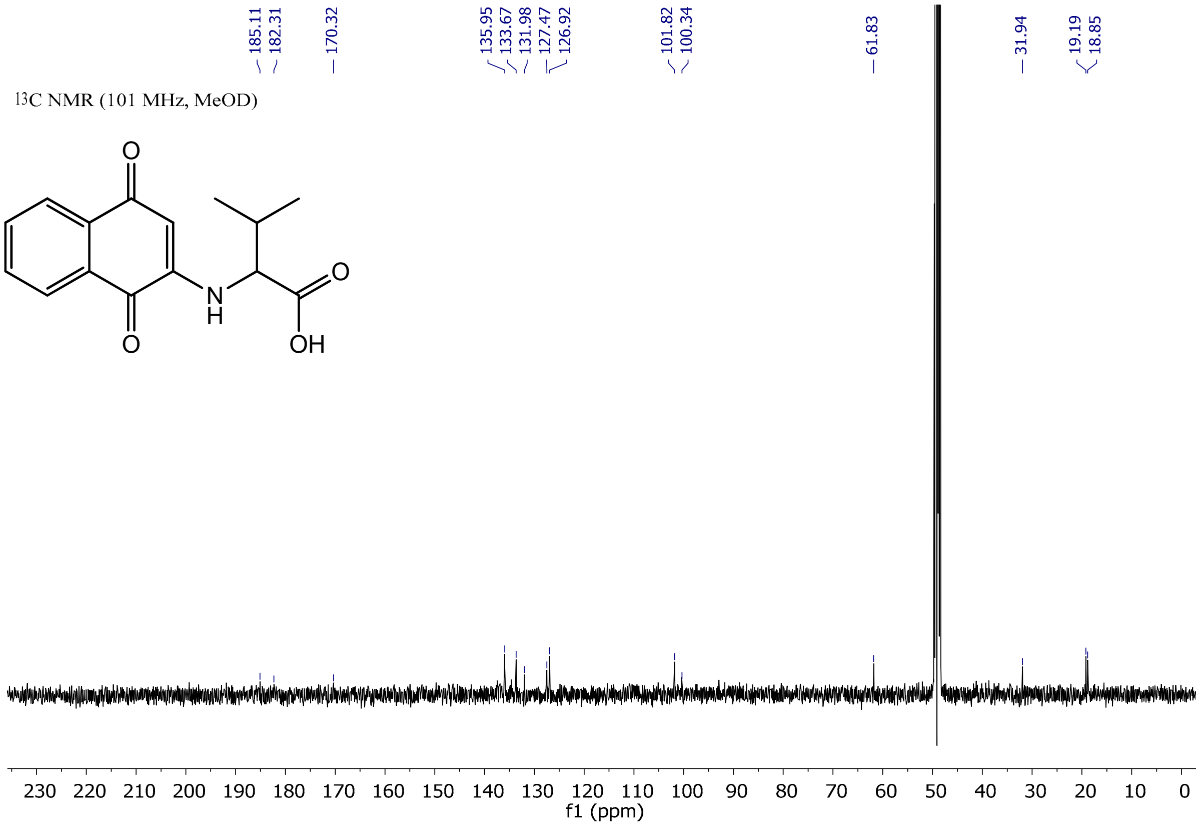

Figure S8. NMR spectrum of 2-((1,4-dioxo-1,4-dihydronaphthalen-2-yl)amino)-3-methylbutanoic acid (3b), ^{1}H (**A**) and ^{13}C (**B**).

A

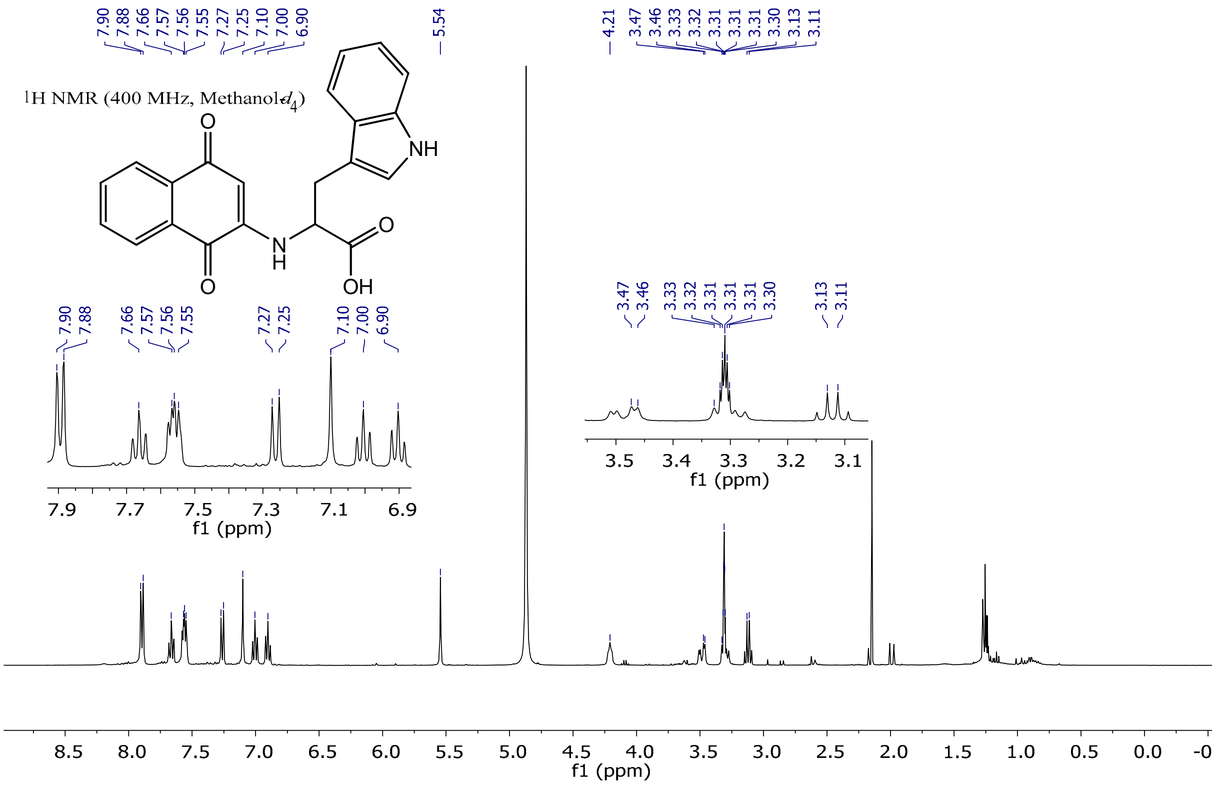

B

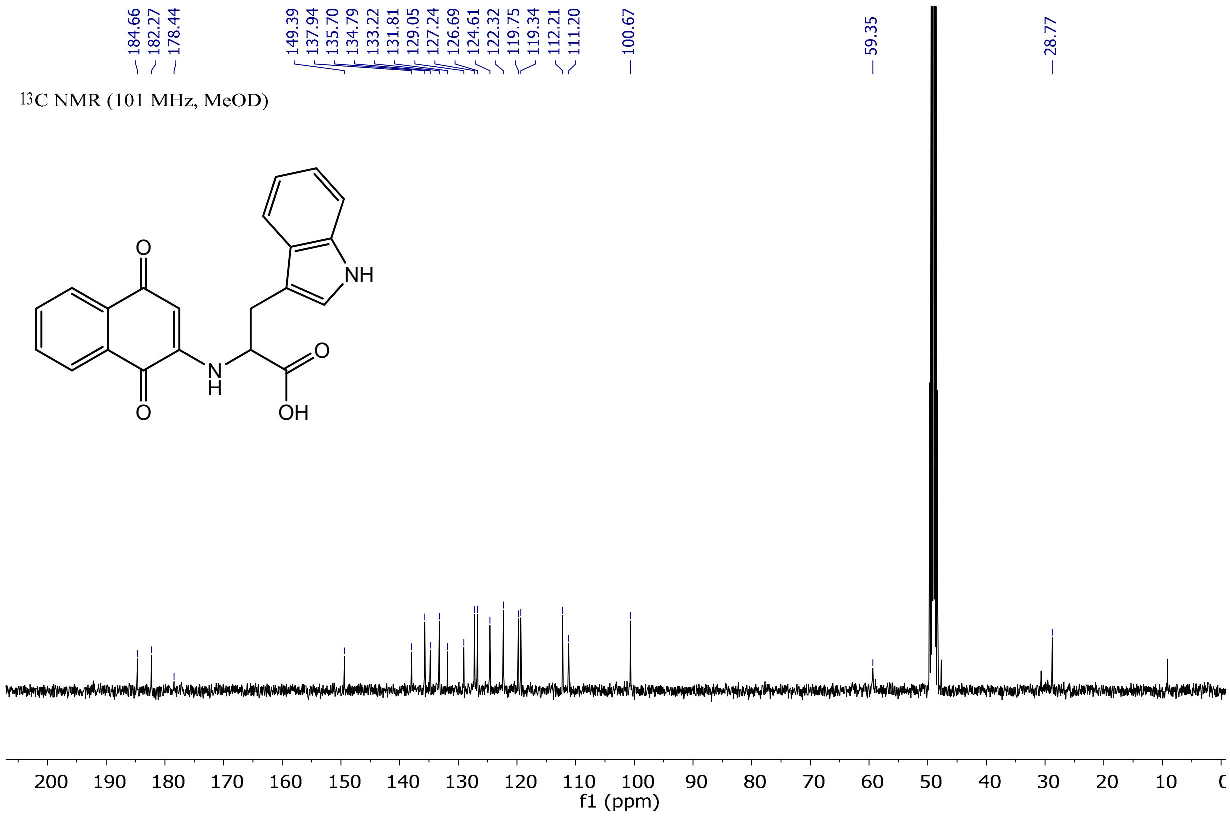

Figure S9. NMR spectrum of 2-((1,4-dioxo-1,4-dihidronaphthalen-2-yl)amino)-3-(1*H*-indol-3-yl)propanoic acid (3c), ^{1}H (**A**) and ^{13}C (**B**).

Amino acids-2,3-dichloronaphthoquinone derivatives:

A

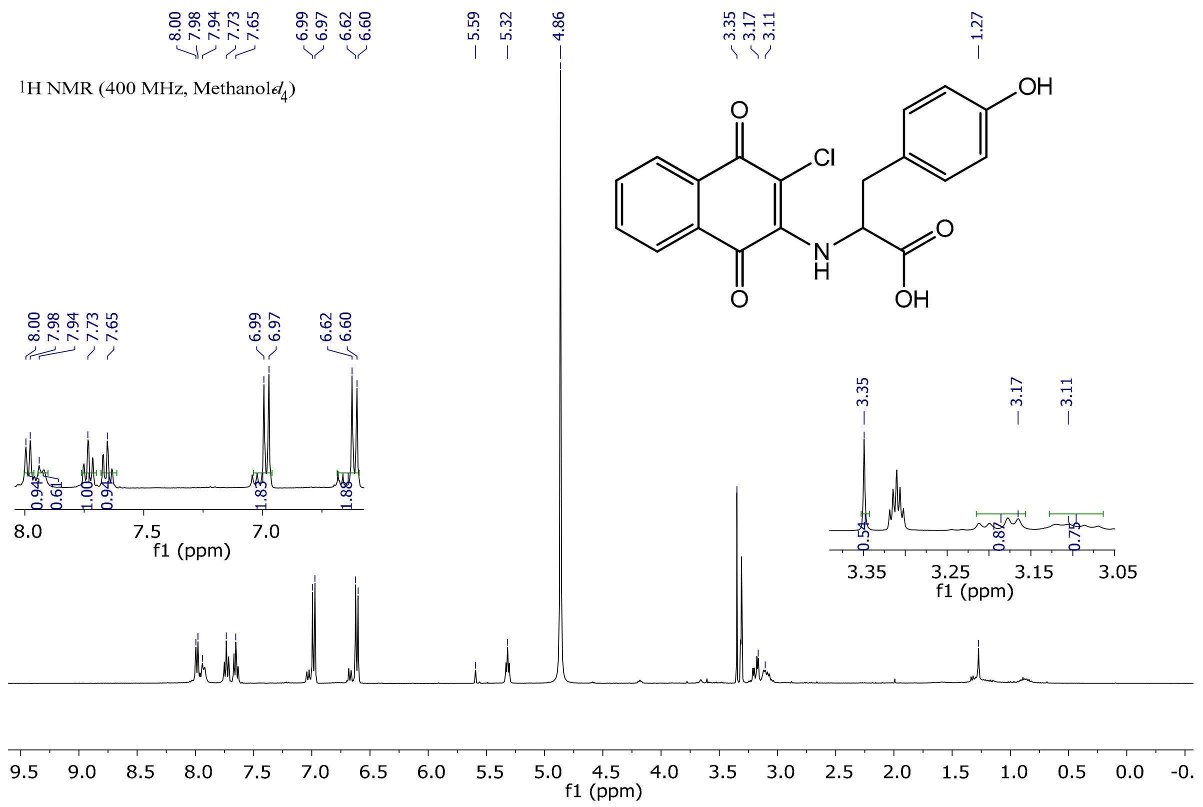

B

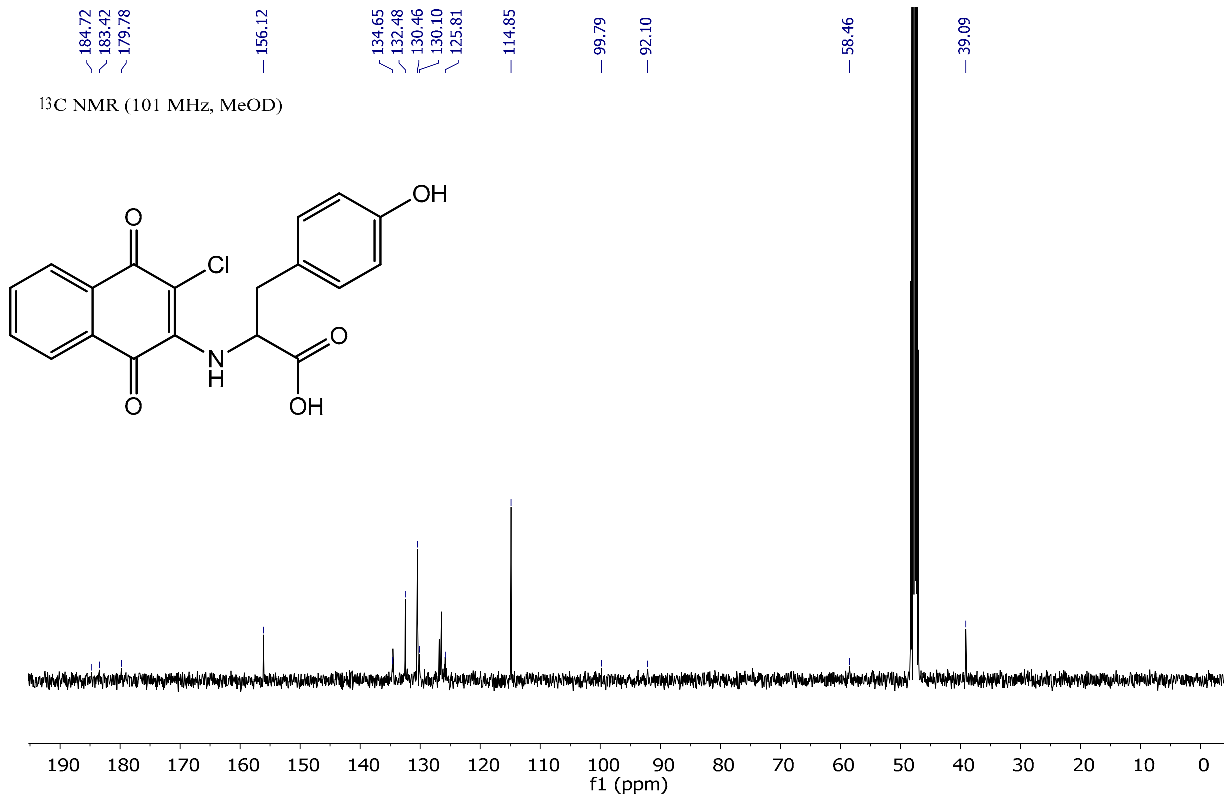

Figure S10. NMR spectrum of 2-((3-chloro-1,4-dioxo-1,4-dihydronaphthalen-2-yl)amino)-3-(4-hydroxyphenyl)propanoic acid (4a), ^{1}H (**A**) and ^{13}C (**B**).

A

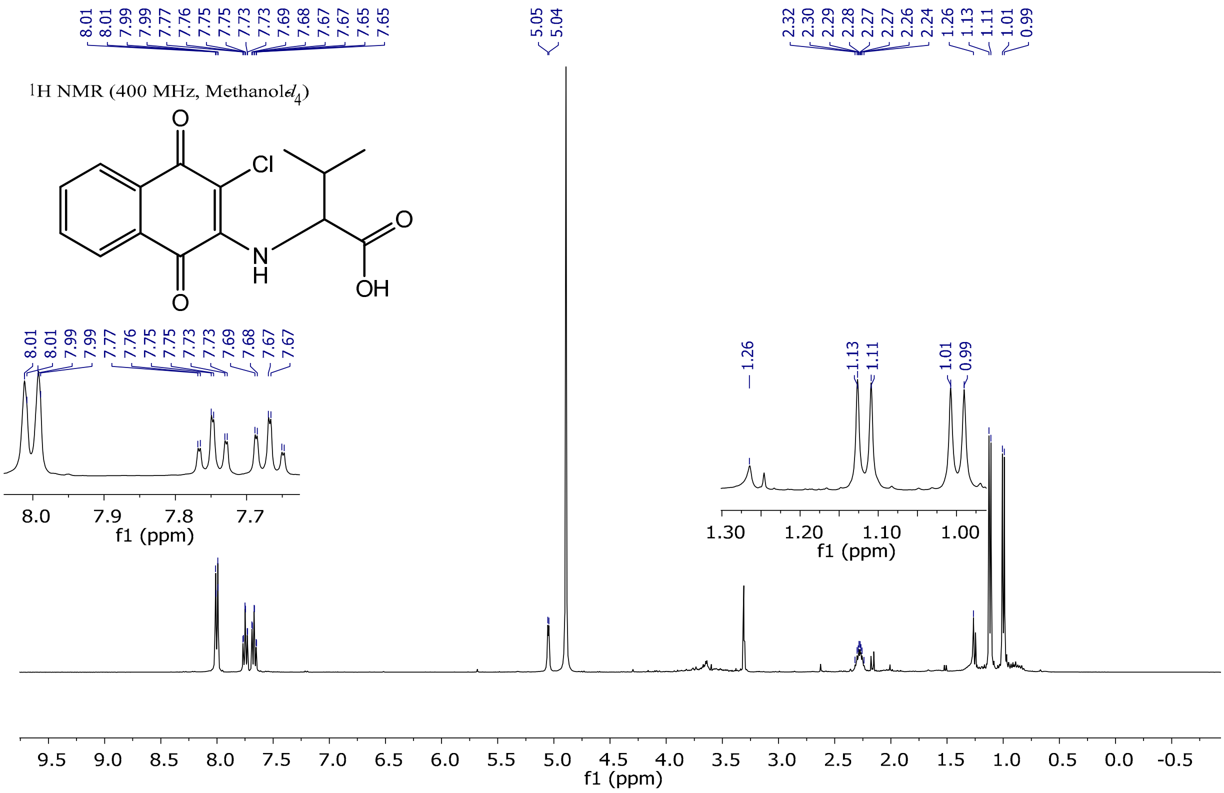

B

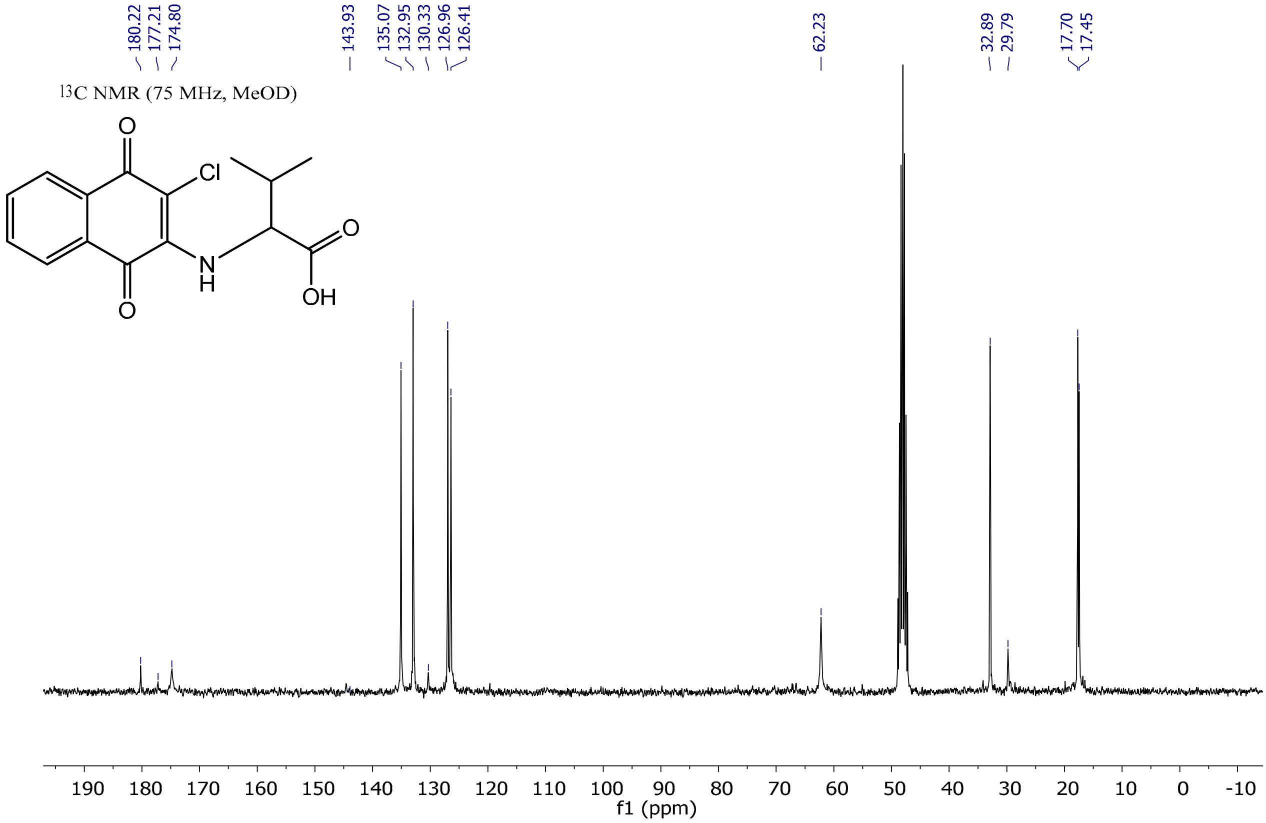

Figure S11. NMR spectrum of 2-((3-chloro-1,4-dioxo-1,4-dihydronaphthalen-2-yl)amino)-3-methylbutanoic acid (4b), ^{1}H (**A**) and ^{13}C (**B**).

A

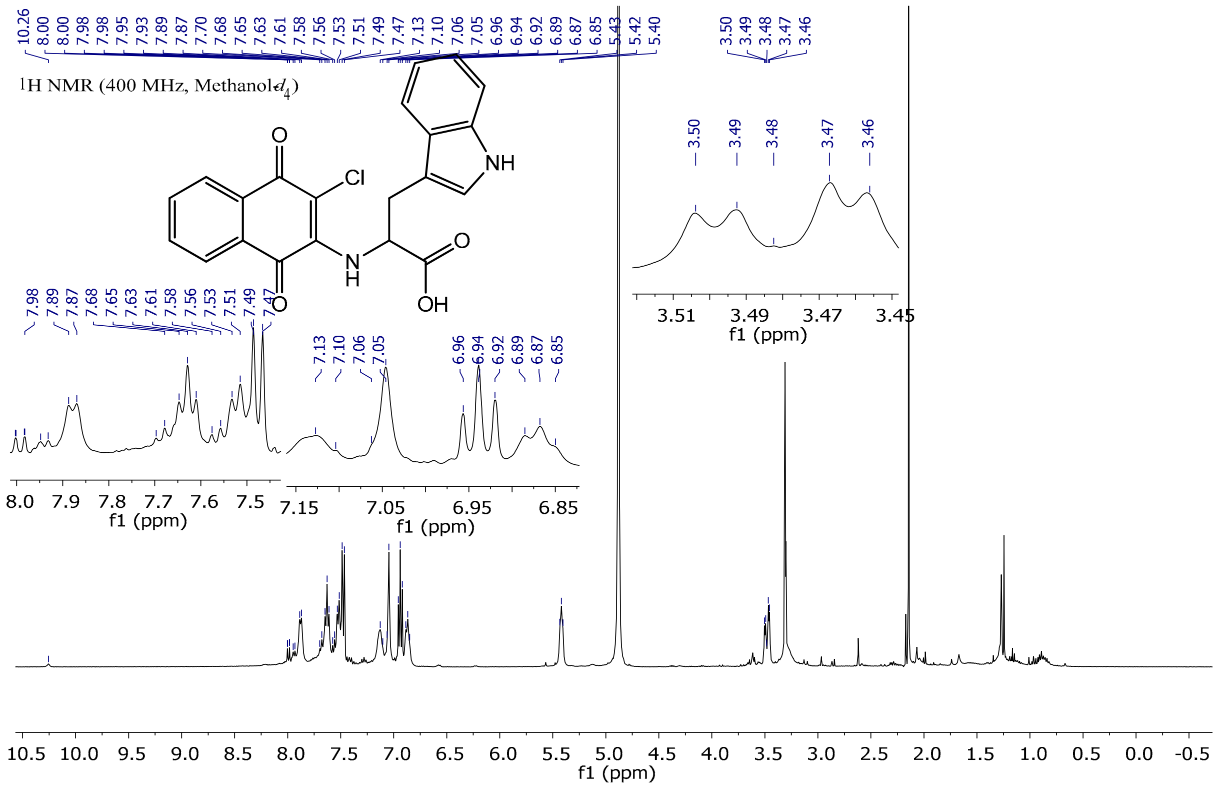

B

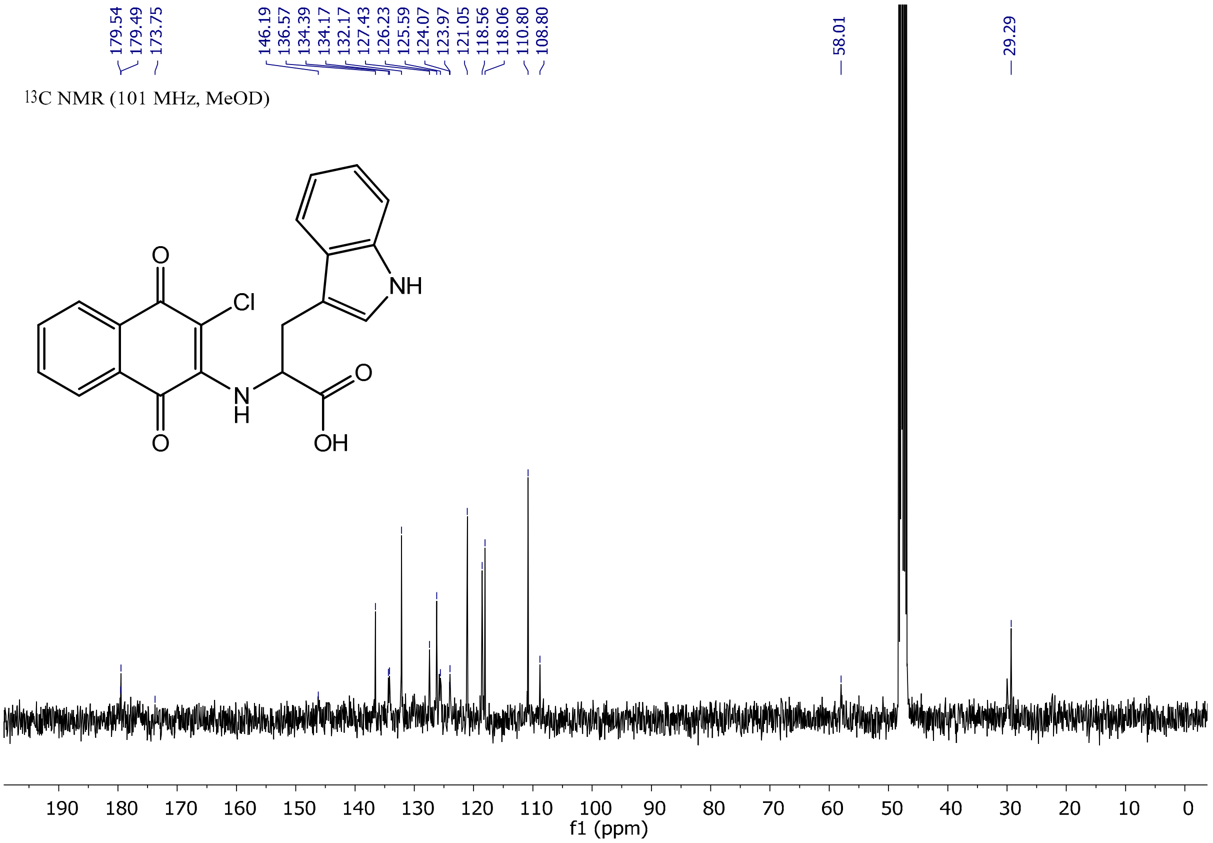

Figure S12. NMR spectrum of 2-((3-chloro-1,4-dioxo-1,4-dihydronaphthalen-2-yl)amino)-3-(1H-indol-3-yl)propanoic acid (4c), ^{1}H (**A**) and ^{13}C (**B**).

3. Mass Spectrometry Characterization

Amino acids-1,4-naphthoquinone derivatives:

**Data Filename**	Nfa Tyr.d	**Sample Name**	Nfa Tyr
**Sample Type**	Sample	**Position**	P1-B4
**Instrument Name**	LC QTOF-LANCIC	**User Name**	
**Acq Method**	Ine directa pos.m	**Acquired Time**	3/15/2019 1:12:48 PM
**IRM Calibration Status**	Success	**DA Method**	Everardo ESI MS.m
**Comment**			

**Sample Group**		**Info.**	
**Stream Name**	LC 1	**Acquisition SW Version**	6200 series TOF/6500 series Q-TOF B.06.01 (B6172 SP1)

User Spectra

Fragmentor Voltage	Collision Energy	Ionization Mode
150	0	ESI

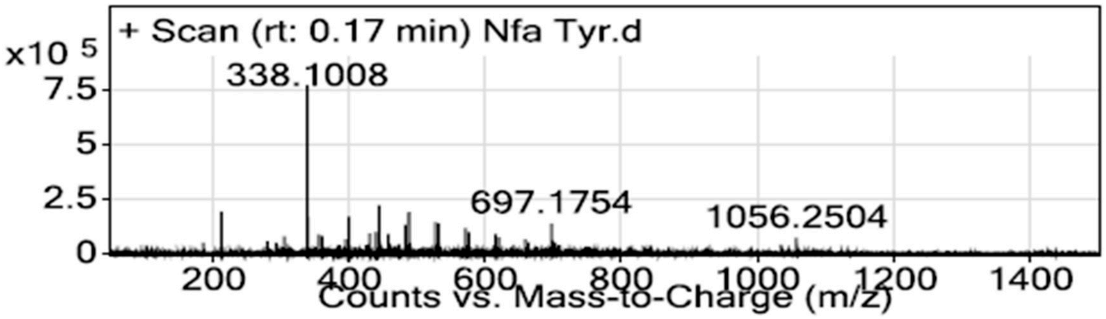

Figure S13. MS spectrum of 2-((1,4-dioxo-1,4-dihydronphthalen-2-yl)amino)-3-(4-hydroxyphenyl) propanoic acid (3a).

**Data Filename**	Nfa Va.d	**Sample Name**	Nfa Va
**Sample Type**	Sample	**Position**	P1-B5
**Instrument Name**	LC QTOF-LANCIC	**User Name**	
**Acq Method**	Ine directa pos.m	**Acquired Time**	3/15/2019 1:17:54 PM
**IRM Calibration Status**	Success	**DA Method**	Everardo ESI MS.m
**Comment**			

**Sample Group**		**Info.**	
**Stream Name**	LC 1	**Acquisition SW Version**	6200 series TOF/6500 series Q-TOF B.06.01 (B6172 SP1)

User Spectra

Fragmentor Voltage	Collision Energy	Ionization Mode
150	0	ESI

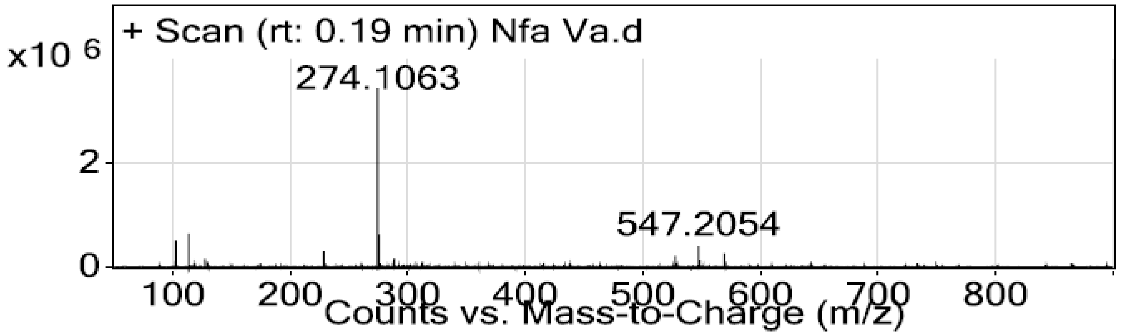

Figure S14. MS spectrum of 2-((1,4-dioxo-1,4-dihydronaphthalen-2-yl)amino)-3-methylbutanoic acid (3b).

**Data Filename**	Nfa Trp.d	**Sample Name**	Nfa Trp
**Sample Type**	Sample	**Position**	P1-B6
**Instrument Name**	LC QTOF-LANCIC	**User Name**	
**Acq Method**	Ine directa pos.m	**Acquired Time**	3/15/2019 1:22:59 PM
**IRM Calibration Status**	Success	**DA Method**	Everardo ESI MS.m
**Comment**			

**Sample Group**		**Info.**	
**Stream Name**	LC 1	**Acquisition SW Version**	6200 series TOF/6500 series Q-TOF B.06.01 (B6172 SP1)

User Spectra

Fragmentor Voltage	Collision Energy	Ionization Mode
150	0	ESI

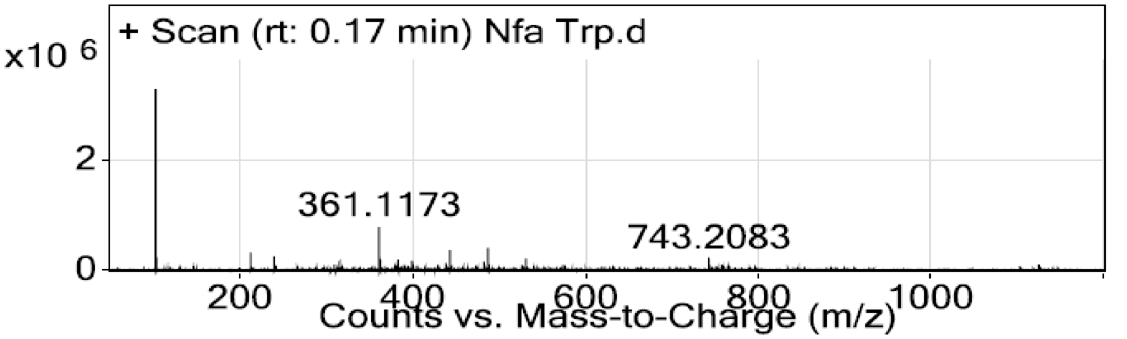

Figure S15. MS spectrum of 2-((1,4-dioxo-1,4-dihidronaphthalen-2-yl)amino)-3-(1*H*-indol-3-yl)propanoic acid (3c).

Amino acids-2,3-dichloronaphthoquinone derivatives:

Data:U-1240 NCI Tyr
Sample Name:Dr. Braulio Rodriguez / Ernesto Rivera
Description:
Ionization Mode:ESI+
History:Determine m/z[Peak Detect[Centroid,30,Area];Correct Base[1.0%];Smooth..

Acquired:3/12/2019 3:12:11 PM
Operator:AccuTOF
Mass Calibration data:Cal Peg 600
Created:3/12/2019 3:43:36 PM
Created by:AccuTOF

Charge number:1 Tolerance:50.00(mmu) Unsaturation Number:1.0 .. 100.0 (Fra...
Element:^{12}C:1 .. 19, ^{1}H:1 .. 15, ^{35}Cl:1 .. 1, ^{14}N:1 .. 1, ^{16}O:1 .. 5

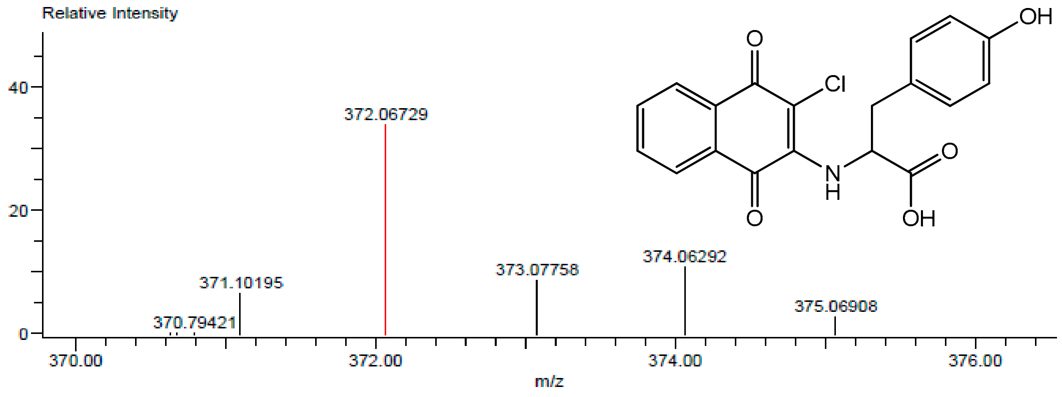

Mass	Intensity	Calc. Mass	Mass Difference (mmu)	Mass Difference (ppm)	Possible Formula
372.06729	35830.25	372.06387	3.42	9.19	$^{12}C_{19}{}^{1}H_{15}{}^{35}Cl_{1}{}^{14}N_{1}{}^{16}O_{5}$

Figure S16. MS spectrum of 2-((3-chloro-1,4-dioxo-1,4-dihydronaphthalen-2-yl)amino)-3-(4-hydroxyphenyl)propanoic acid (4a).

**Data Filename**	NCl Va.d	**Sample Name**	NCl Va
**Sample Type**	Sample	**Position**	P1-B2
**Instrument Name**	LC QTOF-LANCIC	**User Name**	
**Acq Method**	Ine directa pos.m	**Acquired Time**	3/15/2019 1:02:38 PM
**IRM Calibration Status**	Success	**DA Method**	Everardo ESI MS.m
**Comment**			

**Sample Group**		**Info.**	
**Stream Name**	LC 1	**Acquisition SW Version**	6200 series TOF/6500 series Q-TOF B.06.01 (B6172 SP1)

User Spectra

Fragmentor Voltage	Collision Energy	Ionization Mode
150	0	ESI

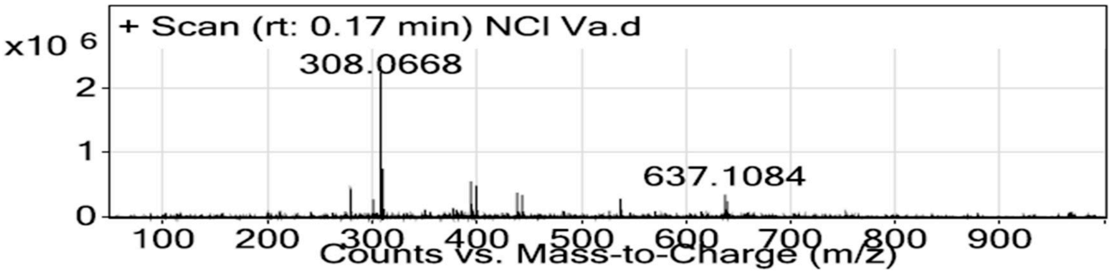

Figure S17. MS spectrum of 2-((3-chloro-1,4-dioxo-1,4-dihydronaphthalen-2-yl)amino)-3-methylbutanoic acid (4b).

**Data Filename**	NCl Trp.d	**Sample Name**	NCl Trp
**Sample Type**	Sample	**Position**	P1-B3
**Instrument Name**	LC QTOF-LANCIC	**User Name**	
**Acq Method**	Ine directa pos.m	**Acquired Time**	3/15/2019 1:07:43 PM
**IRM Calibration Status**	Success	**DA Method**	Everardo ESI MS.m
**Comment**			

**Sample Group**		**Info.**	
**Stream Name**	LC 1	**Acquisition SW Version**	6200 series TOF/6500 series Q-TOF B.06.01 (B6172 SP1)

User Spectra

Fragmentor Voltage	Collision Energy	Ionization Mode
150	0	ESI

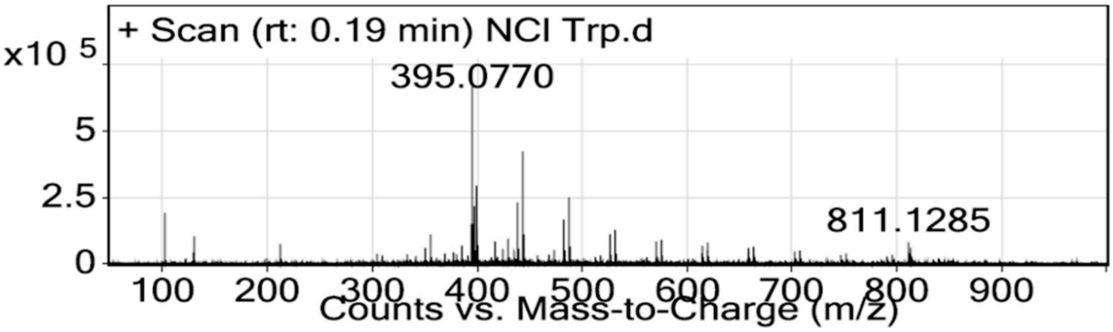

Figure S18. MS spectrum of 2-((3-chloro-1,4-dioxo-1,4-dihydronaphthalen-2-yl)amino)-3-(1H-indol-3-yl)propanoic acid (4c).

4. EPR Characterization

Compound 2-((1,4-dioxo-1,4-dihydronaphthalen-2-yl)amino)-4-(methylthio)butanoic acid

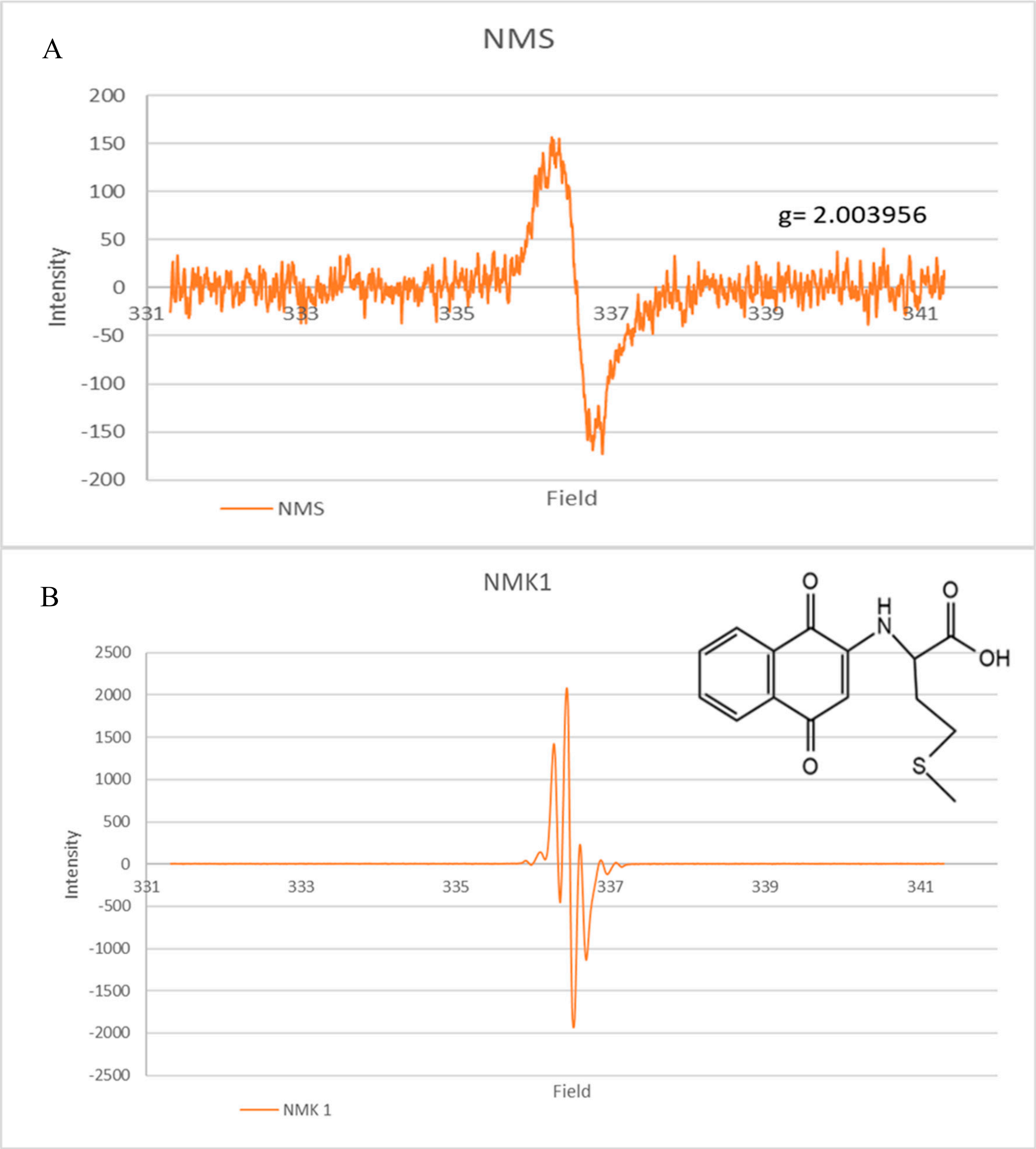

Figure S19. EPR spectrum of 2-((1,4-dioxo-1,4-dihydronaphthalen-2-yl)amino)-4-(methylthio)butanoic acid, in solid state (A) and KOH 1N solution (B).
